# Investigating the role of ultrasound-based shear wave elastography in kidney transplanted patients: correlation between non-invasive fibrosis detection, kidney dysfunction and biopsy results—a systematic review and meta-analysis

**DOI:** 10.1007/s40620-023-01856-w

**Published:** 2024-03-01

**Authors:** Teodóra Filipov, Brigitta Teutsch, Anett Szabó, Attila Forintos, Júlia Ács, Alex Váradi, Péter Hegyi, Tibor Szarvas, Nándor Ács, Péter Nyirády, Pál Ákos Deák

**Affiliations:** 1 Department of Interventional Radiology, Heart and Vascular Center, Faculty of Medicine, Semmelweis University, Határőr ut 18, 1122 Budapest, Hungary; 2 Centre for Translational Medicine, Semmelweis University, Budapest, Hungary; 3 Institute for Translational Medicine, Medical School University of Pécs, Pécs, Hungary; 4 Institute of Pancreatic Diseases, Semmelweis University, Budapest, Hungary; 5 Department of Urology, Faculty of Medicine, Semmelweis University, Budapest, Hungary; 6 Department of Laboratory Medicine, Medical School, University of Pécs, Pécs, Hungary; 7 Department of Metagenomics, University of Debrecen, Debrecen, Hungary; 8 Department of Urology, University of Duisburg-Essen and German Cancer Consortium (DKTK)-University Hospital Essen, Essen, Germany; 9 Department of Obstetrics and Gynecology, Faculty of Medicine, Semmelweis University, Budapest, Hungary

**Keywords:** Sonoelastography, Shear wave elastography, Renal transplantation, Biopsy

## Abstract

**Introduction:**

Interstitial fibrosis and tubular atrophy are leading causes of renal allograft failure. Shear wave elastography could be a promising noninvasive method for providing information on the state of the kidney, with specific regard to fibrosis but currently available data in the literature are controversial. Our study aimed to analyze the correlation between shear wave elastography and various kidney dysfunction measures.

**Methods:**

This review was registered on PROSPERO (CRD42021283152). We systematically searched three major databases (MEDLINE, Embase, and CENTRAL) for articles concerning renal transplant recipients, shear wave elastography, fibrosis, and kidney dysfunction. Meta-analytical calculations for pooled Pearson and Spearman correlation coefficients (*r*) were interpreted with 95% confidence intervals (CIs). Heterogeneity was tested with Cochran’s *Q* test. *I*^2^ statistic and 95% CI were reported as a measurement of between-study heterogeneity. Study quality was assessed with the QUADAS2 tool.

**Results:**

In total, 16 studies were included in our meta-analysis. Results showed a moderate correlation between kidney stiffness and interstitial fibrosis and tubular atrophy, graded according to BANFF classification, on biopsy findings for pooled Pearson (*r* = 0.48; CI: 0.20, 0.69; *I*^2^ = 84%) and Spearman correlations (*r* = 0.57; CI: 0.35, 0.72; *I*^2^ = 74%). When compared to kidney dysfunction parameters, we found a moderate correlation between shear wave elastography and resistive index (*r* = 0.34 CI: 0.13, 0.51; *I*^2^ = 67%) and between shear wave elastography and estimated Glomerular Filtration Rate (eGFR) (*r* = -0.65; CI: − 0.81, − 0.40; *I*^2^ = 73%). All our outcomes had marked heterogeneity.

**Conclusion:**

Our results showed a moderate correlation between kidney stiffness measured by shear wave elastography and biopsy results. While noninvasive assessment of kidney fibrosis after transplantation is an important clinical goal, there is insufficient evidence to support the use of elastography over the performance of a kidney biopsy.

**Graphical abstract:**

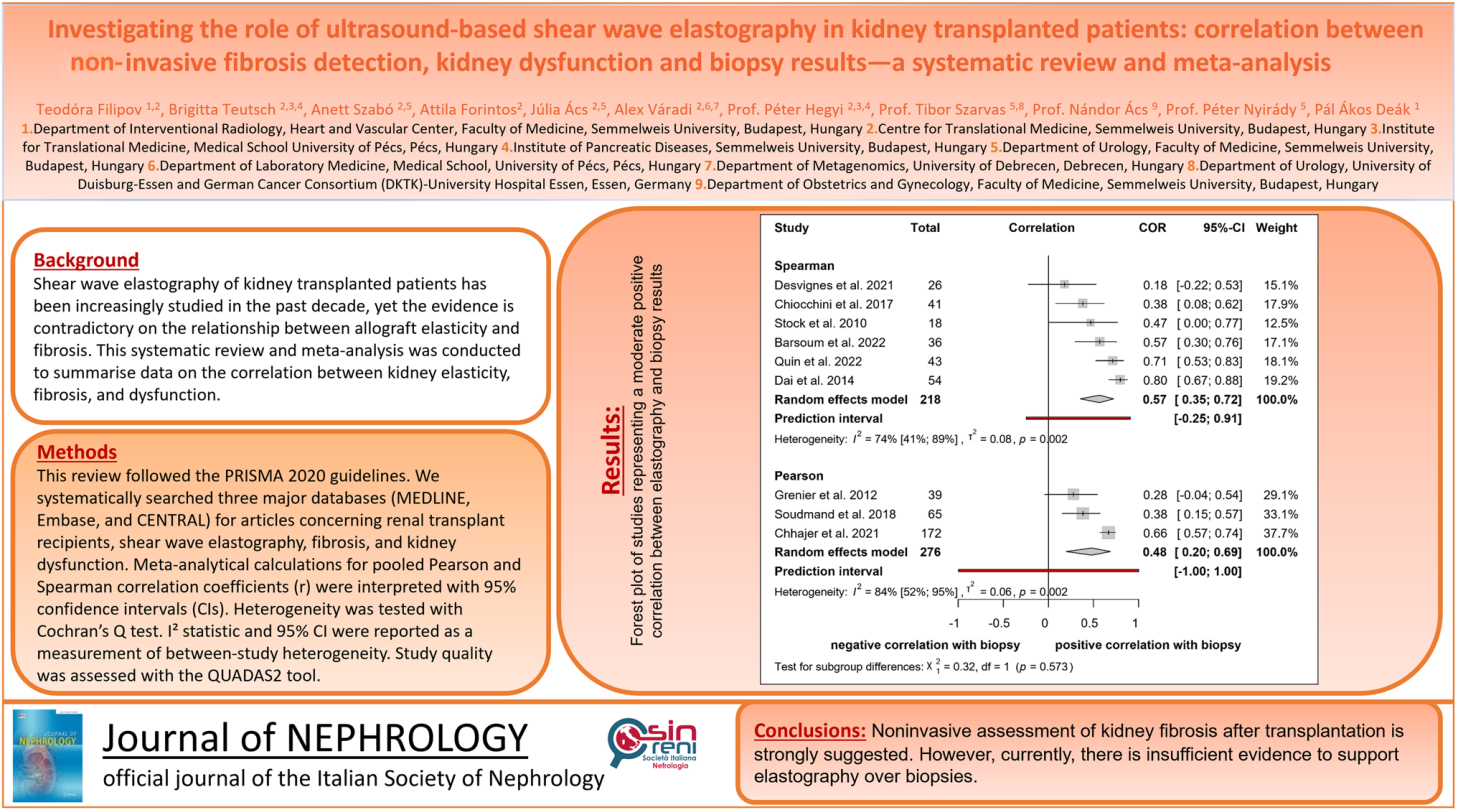

**Supplementary Information:**

The online version contains supplementary material available at 10.1007/s40620-023-01856-w.

## Introduction

It is known that pathways of renal graft dysfunction ultimately lead to a common endpoint: fibrosis. Therefore, interstitial fibrosis and tubular atrophy are considered the most common causes of allograft loss [1]. Interstitial fibrosis and tubular atrophy start early after transplantation and are rooted in multiple causes, including acute or persistent subclinical rejection and ischemia–reperfusion injury [2]. These conditions result in increased serum creatinine, decreased estimated glomerular filtration rate (eGFR), proteinuria, and hypertension [1, 3].

It is crucial to monitor allograft function after transplantation. Currently, information provided by kidney biopsy can clarify the diagnosis of graft dysfunction and serve as a guide to clinical management [4]. Although biopsies are considered safe, they hold risks, and major complications include arteriovenous fistula, hemorrhage and rarely even graft loss [5–8]. Additionally, biopsies cannot be performed in the presence of severe thrombocytopenia, anticoagulant usage, severe hypertension, bacteremia, or uncorrectable coagulopathy [9, 10].

In the past decade, options to minimize the need for invasive procedures have been explored. Ultrasound-based elastography seems to be a promising modality for assessing the state of the kidney, as changes in tissue elasticity are linked to pathological processes [11].

Shear wave elastography is a form of dynamic elastography that provides information on the elastic properties of tissues by measuring shear-wave speed [12]. It has already proven to be useful in the detection of liver fibrosis; broadening of its application to other organs, including the breast, prostate, lymph nodes, thyroid, and kidneys is being tested [12]. However, apart from their highly anisotropic nature, urinary pressure, vascular perfusion, hydronephrosis, and body mass index can affect shear wave elastography results [1]. Many studies have reported the link between kidney elasticity and fibrosis; still, there are conflicting data on this relationship. Some studies observed no correlation between kidney stiffness measured by shear wave elastography and biopsy results [13, 14], while others report that stiffness is positively correlated to fibrosis [10, 15]. Given the controversy, we conducted a meta-analysis to investigate the correlation between shear wave elastography findings, biopsy results, and renal dysfunction parameters in kidney transplant recipients.

## Methods

Our systematic review and meta-analysis is reported following the recommendations of the PRISMA 2020 guidelines [16] (see Supplementary Table S1) while referring to the Cochrane Handbook for Systematic Reviews of Interventions [17]. The protocol of the study was registered on PROSPERO under registration number CRD42021283152.

### Literature search, data sources, and study selection

Our systematic search was conducted on October 17, 2021, in three major medical databases (MEDLINE, Embase, and CENTRAL). On February 15, 2023, we reran our systematic search to identify additional relevant articles. Language or date restrictions were not applied. The search key used in all databases is detailed in the supplementary material (Table S2.). All types of observational studies investigating kidney transplant recipients and reporting the correlation coefficient for shear wave elastography values and kidney dysfunction parameters were found eligible. Kidney dysfunction parameters were defined as fibrosis, resistive index, serum creatinine, and eGFR. Animal studies, reviews, letters, case reports, and studies using transient- or magnetic resonance elastography were excluded.

Two independent review authors (TF and ASz) performed the selection of potentially eligible studies. We used EndNote X9 (Clarivate Analytics) reference manager software in the article selection process. After duplicate removal, selection by title and abstract was followed by the selection of full texts. We measured inter-rater consistency with Cohen's kappa coefficient (κ), calculated after each step. If there were disagreements regarding a study, its eligibility was decided by a third reviewer (BT).

Backward and forward citation searching of all eligible articles was also conducted to identify further articles.

Two authors (TF and AF) collected data from the eligible articles independently. A third reviewer (BT) helped to resolve disagreements. We collected data in pre-defined Excel sheets (Microsoft Corporation). The extracted contents included: study characteristics: first author, publication year, Digital Object Identifier, study design, study location, and the number of patients; and baseline patient data: age, gender, time elapsed since transplantation, alive or deceased donation, and Banff fibrosis scores (if applicable). We also collected technical features of the ultrasound devices, the shear wave elastography technique, and raw data about shear wave elastography and available details on operators. Regarding the outcomes of our study, the correlation coefficients (Pearson's or Spearman's) and corresponding p values between shear wave elastography and kidney dysfunction parameters were calculated. Study authors were contacted if important data were not reported in the articles.

### Study quality evaluation

The risk of bias assessment was carried out independently by two authors (FT, AF) using the QUADAS-2 tool [18], which consists of two parts: concerns about bias and practical applicability. The former was assessed in terms of the following four domains: patient selection, index test, reference standard, and flow and timing; the latter enclosed three elements: patient selection, index test, and reference standard. In case of disagreements about the quality of a study, a third investigator (BT) helped in the decision.

### Strategy for data synthesis

A minimum of three studies per outcome were required to be included in our meta-analysis. Outcomes that did not reach this number were only included in Forest plots for visualization. The statistical analysis of data was carried out using the R programming language (R Core Team, 2019, version 4.1). To calculate random effects estimates for meta-analysis with correlation data we used the *metacor* function of the *meta* v5.5 R package [19].

Using the extracted correlation coefficient (*r*) from each study, we calculated pooled correlation coefficients with 95% confidence intervals (CI) using the random-effects model with the inverse variance weighting method and Restricted Maximum Likelihood method estimator for between-study variance [20]. Before analysis, correlation coefficients had to be transformed into Fisher’s z (*z* = 0.5 log e (1 + r/1-r)) unless the included studies had very large sample sizes [21]. This transformation was automatically performed by *metacor* function, with the sm argument set to "ZCOR". The different types of correlations were not pooled together, since Pearson's product-moment correlation is used when a linear relationship is assumed between two continuous, random variables, and Spearman's rank correlation is used when the relationship of two variables appears to be monotonic, but nonlinear. The correlation strength was ranked as follows: 0.00–0.10 was considered negligible, 0.10–0.39 weak, 0.40–0.69 moderate, 0.70–0.89 strong, and 0.90–1.00 very strong [22]. Results were considered statistically significant if *p* < 0.05. Forest plots were used to graphically summarize the results.

Heterogeneity was tested with Cochran’s *Q* tests and significant heterogeneity was indicated by *p* < 0.1. We report *I*^2^ statistics and their 95% CI, which represent the percentage of total variation across studies due to between-study heterogeneity [23]. According to the Cochrane Handbook for Systematic Reviews of Interventions [17], a rough guide for the interpretation of *I*^*2*^ at 0% to 40% might not be important, 30% to 60% may represent moderate heterogeneity, 50% to 90% may represent substantial heterogeneity, and 75% to 100% significant heterogeneity. Furthermore, where applicable, we reported the prediction intervals (i.e., the expected range of effects of future studies) of pooled estimates as well [24].

The estimation of publication bias was not possible because the number of articles did not reach the minimum of 10 for this assessment.

## Results

### Systematic search and study selection

**Fig. 1:**
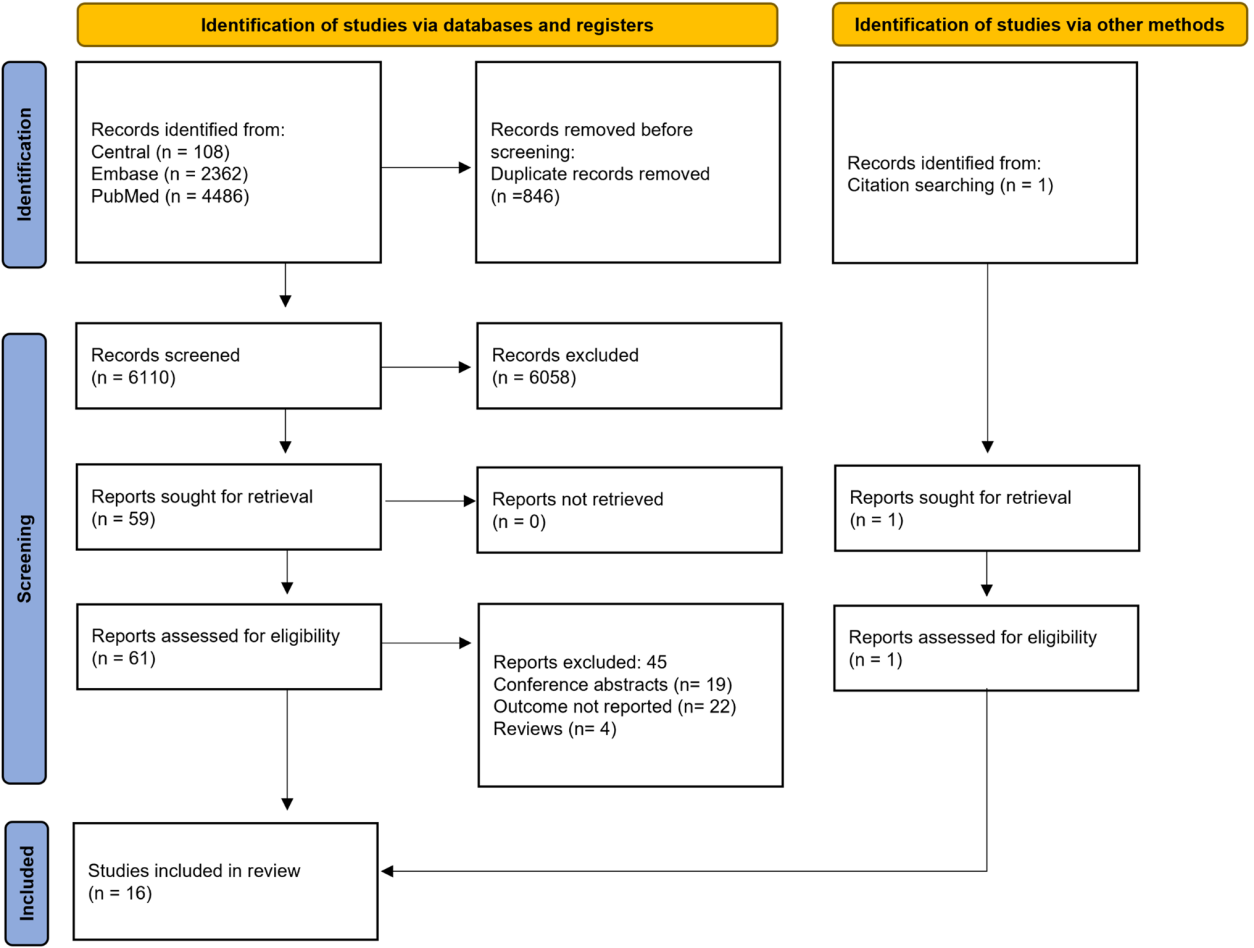
PRISMA 2020 flowchart representing the study selection process

Through our systematic search, we identified a total of 6956 studies. Interrater reliability assessment resulted in a Cohen's kappa of 0.82 and 1.0 for the title and abstract selection and full-text selection, respectively. At the end of the study selection process, 16 studies [10, 15, 25–38] were included in the meta-analysis, one of which [25] was added during reference searching. A more detailed outline of our selection process is depicted in Fig. 1.

### Basic characteristics of included studies

Baseline characteristics of the populations and technical features of the included studies are detailed in Tables 1 and 2, respectively. The articles were published from 2010 to 2022, and the total number of patients assessed in this meta-analysis was 931. One article [28] reported on a pediatric population; all others examined adults. The publications included participants from 9 countries in total. Two articles [31, 38] were not written in English; for their translation we requested the help of a translator.

**Table 1:** Main characteristics of included studies

Study	Correlation coefficient	Study period	Country	No. of patients	Age (year)		Sex (Female, %)	Donor living/deceased	Time elapsed since Tx (m) mean (range)	Mean creatinine (mg/dl)	Mean eGFR
					Mean ± SD	Range					
Agrawal et al. 2021 [25]	Pearson's	10.2018–07.2020	India	40	39.2 ± 11.7	21–61	15	N/A	22 (12–26)	N/A	63.3
Barsoum et al. 2022 [36]	Spearman’s	02.2021–08.2021	Egypt	36	N/A	N/A	N/A	36/0	4.33 (2–8)	2.76	N/A
Chhajer et al. 2021 [15]	Pearson's	01.2017–03.2019	India	172	43.8	9–64	26.1	N/A	23.9 (3–180)	N/A	N/A
Chiocchini et al. 2017 [26]	Spearman's	N/A	Italy	41	52 ± 16	N/A	38.1	1/40	9^a^ (1–288)	3.6	24.9
Dai et al. 2014 [27]	Spearman's	10.2010- 07.2013	China	54	38 ± 10	20–65	N/A	N/A	N/A	N/A	N/A
Desvignes et al. 2021 [28]	Spearman's	N/A	France	26	N/A	4 m-18y	38.5	N/A	N/A	N/A	N/A
Grenier et.al. 2012 [30]	Pearson's	01.2010–05.2010	France	39	51^a^	18.5–69.9	51	47/2	26.6^a^ (0.3–214.3)	1.9	34
Ghonge et al. 2018 [29]	Pearson's	10.2014–3.2016	India	60	40.8 ± 11.3	20–73	15	60/0	26.8 (0.3–160)	0.76/1.9/ 3.9^b^	83.1/ 47.7/31.3^b^
He et al. 2014 [31]	Spearman's	12.2011–03.2013	China	102	38 ± 12	18–64	32.3	N/A	31 (1–120)	N/A	N/A
Järv et al. 2019 [32]	Spearman's	03.2017–11.2017	Estonia	100	53.3 ± 9.4	22–79	41	N/A	N/A	N/A	53.8
Quin et al. 2022	Pearson’s & Spearman’s	09.2020–08.2021	China	43	43^a^	38–56	11.6	7/36	48^a^ (28–64)	2.35^a^	31.2
Soudmand et al. 2018 [10]	Pearson's	N/A	Turkey	65	38.8 ± 14	24.8–52.8	23.1	51/14	N/A	2.4	N/A
Stock et al. 2010 [33]	Spearman's	03.2009- 06.2009	Germany	18	54.3 ± 14.6	26–76	27.8	2/16	22^a^ (4.4–54.3)	2.6	28
Tukhbatullin et al. 2017 [34]	Pearson's	02.2015–05.2017	Russia	32	42.9 ± 2.4	N/A	N/A	N/A	N/A	N/A	N/A
Wang et al. 2017 [35]	Pearson's	N/A	Taiwan	40	45.3	21–68	35	27/13	N/A	3.9	27.6
Yang et al. 2022 [38]	Spearman’s	03.2021–11.2021	China	63	45^a^	32–52	49.2	N/A	38^a^ (12–90)	2.4^a^	N/A

^a^parameters represented as median, ^b^: stable group/acute dysfunction group/chronic dysfunction group

*eGFR* estimated glomerular filtration rate, *m* months, *mg/dl* milligrams/deciliter, *N/A* not available, *SD* standard deviation, *Tx* transplantation

**Table 2:** Technical attributes of elastography in included studies

Study	Operators		Technical attributes			ROI		Elastography evaluation	
	No	Experience (y)	Device	Manufacturer	Transducer	Renal compartment	Location	Technique	U
Agrawal et al. 2021 [25]	N/A	N/A	iU22	Philips Healthcare	C5-1 convex (5–1 MHz)	N/A	Upper, middle, lower pole	SWE	kPa
Barsoum et al. 2022 [36]	N/A	N/A	Aplio 500	TOSHIBA	6C1 curvilinear (B-mode)/ 14L5 linear (SWE)	N/A	N/A	SWE	kPa
Chhajer et al. 2021 [15]	N/A	N/A	Logiq E9	GE Healthcare	N/A	N/A	Upper, middle, lower pole	SWE	kPa
Chiocchini et al. 2017 [26]	2	5	iU22	Philips Healthcare	C5-1 convex	N/A	Middle third	SWE	kPa
Dai et al. 2014 [27]	N/A	N/A	Acuson S2000	Siemens Healthineers	4C1 convex (2–4 MHz)	cortex	Upper, middle, lower pole	p-SWE	m/s
Desvignes et al. 2021 [28]	4	7–25	Aixplorer®	SuperSonic Imagine	Convex low frequency (2–5 MHz)	cortex	Lower pole	2D-SWE	kPa
Grenier et.al. 2012 [30]	2	N/A	Aixplorer®	SuperSonic Imagine	SC6-1 convex (3.5 MHz)	cortex and medulla	N/A	2D-SWE	kPa
Ghonge et al. 2018 [29]	1	15	EPIQ-7G	Philips Healthcare	C5–1 convex	N/A	Upper, lower, midinterpolar	p-SWE	kPa
He et al. 2014 [31]	2	N/A	Acuson S2000	Siemens Healthineers	4C1 curved linear array (1,75-4 MHz)	N/A	Middle third	p-SWE	m/s
Järv et al. 2019 [32]	2	> 20	Affiniti 70	Philips Healthcare	C5-1 convex (5–1 MHz)	cortex	Upper, lower pole	SWE	kPa
Quin et al. 2022	1	> 10	MyLab 8Exp	Esaote SpA	C1-8 convex	cortex	N/A	P-SWE	kPa
Soudmand et al. 2018 [10]	1	N/A	Acuson S2000	Siemens Healthineers	6C1 curvilinear	cortex	N/A	p-SWE	m/s
Stock et al. 2010 [33]	3	N/A	Acuson S2000	Siemens Healthineers	curved array (4–1 MHz)	N/A	Upper, middle, lower pole	p-SWE	m/s
Tukhbatullin et al. 2017 [34]	N/A	N/A	Aixplorer®	SuperSonic Imagine	convex (1–6 MHz)	N/A	Upper, lower pole	2D-SWE	kPa
Wang et al. 2017 [35]	1	N/A	Acuson S3000	Siemens Healthineers	linear (4–9 MHz)	cortex	N/A	p-SWE	m/s
Yang et al. 2022 [38]	N/A	N/A	Voluson E20	GE Healthcare	C6-1 curvilinear (B-mode)/ L2-9 linear (SWE)	cortex	N/A	SWE	kPa

*kPa* kilopascal*, MHz* megahertz, *m/*s millimeters/second, *N/A* not available, *p-SWE* point-shear wave elastography, *SWE* shear wave elastography, *y* year

Study populations were quite heterogeneous regarding age (range: 4 m-79y), gender (15–49.2% females), and kidney function. Study inclusion criteria also varied, but shared the same basis of including renal transplants for renal ultrasound examination. Some articles included patients with suspected pathology, while others included stable patients or a mix of both.

### Risk of bias assessment and concerns about applicability

Of the four [26–28, 33] articles calculating the correlation between shear wave elastography and histopathology using Spearman's correlation, only two [27, 33] did not state whether the index test and reference standard were interpreted blindly. Regarding resistive index, concerns about bias were high in the domains of index test and reference standard, as shear wave elastography and Doppler ultrasound were performed in one sitting by the same radiologist; therefore, blinded interpretation was not possible. However, one article [29] stated that ultrasound examinations were performed blinded to clinical data. The overall risk of bias for this outcome is therefore high, but we considered concerns of applicability to be low.

The outcomes concerning laboratory parameters were harder to assess because laboratory test results were not detailed in the articles. However, most articles [15, 26, 29, 31, 32] stated that shear wave elastography was performed blinded to clinical data.

To sum up, the overall risk of bias varied from low to high concerning different outcomes, and we considered concerns about applicability to be low. Tables and diagrams detailing the results of the assessment of the risk of bias and applicability are to be found in the supplementary material (Table S3–S10, Figure S1–S8).

### Quantitative and qualitative synthesis

#### Correlation between elastography and biopsy results

**Fig. 2:**
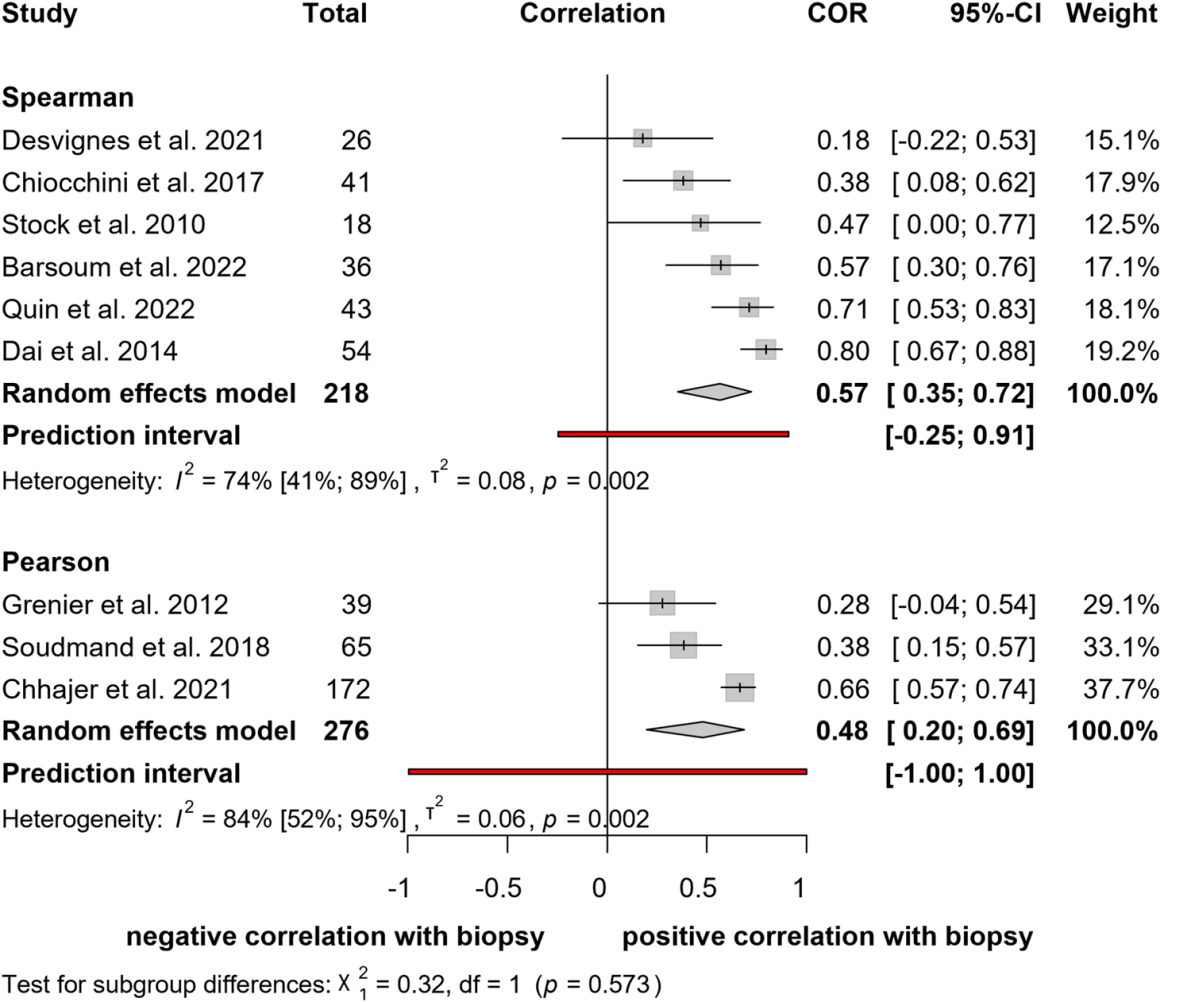
Forest plot of studies representing a moderate positive correlation between elastography and biopsy results

Nine [10, 15, 26–28, 30, 33, 36, 37] studies, with a total of 494 patients, included calculated correlation coefficients between stiffness measured by shear wave elastography and fibrosis according to histopathology (Fig. 2). The pooled results showed a moderate positive correlation for Pearson (*r* = 0.48; CI: 0.20, 0.69) and Spearman correlation coefficients (*r* = 0.57; CI: 0.35, 0.72). Heterogeneity test results showed marked heterogeneity among the studies (*I*^*2*^ = 84%; *p* = 0.002 and *I*^*2*^ = 74%; *p* = 0.002).

Determining fibrosis according to histopathology was based on the BANFF classification, defined as interstitial fibrosis and tubular atrophy lesion score (“ci” + ”ct”) and graded from 0-III. Desvignes et al. [28] calculated correlations with the BANFF “ci” lesion score only.

#### Correlation between elastography and arterial Resistive Index

**Fig. 3:**
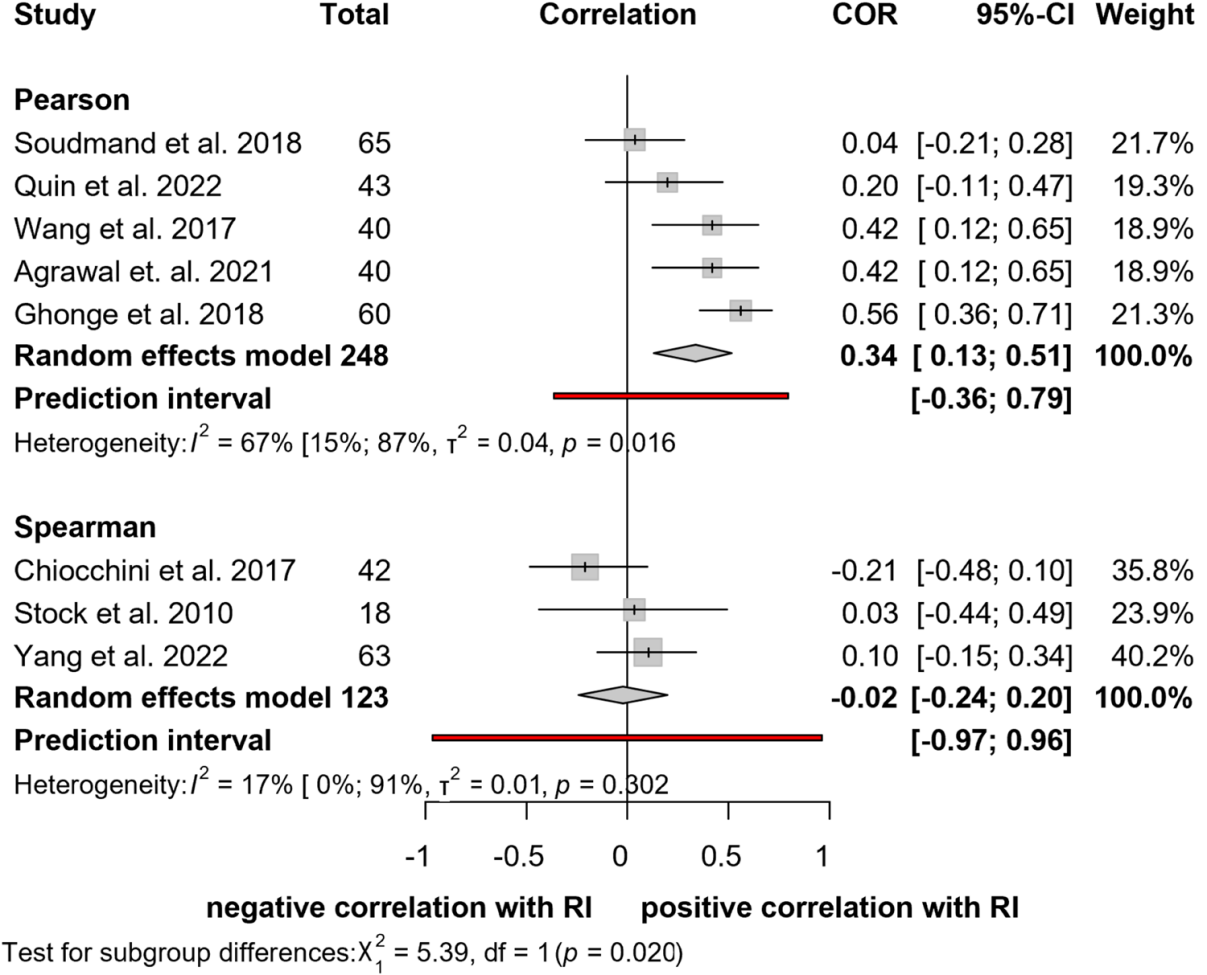
Forest plot of studies representing a weak positive correlation between RI and elastography

Eight [10, 25, 26, 29, 33, 35, 37, 38] studies assessing 371 patients were evaluated with respect to the relationship between shear wave elastography and renal arterial resistive index (Fig. 3). Pooled Pearson's correlation between shear wave elastography and resistive index was weak (*r* = 0.34; CI: 0.13, 0.51). Heterogeneity assessment showed substantial heterogeneity among studies (*I*^*2*^ = 73%; *p* < 0.011). Pooled Spearman’s correlation for the same outcome showed no correlation (*r* = − 0.02; CI:− 0.24, 0.20) and low heterogeneity (*I*^2^ = 17%; *p* = 0.302).

#### Correlation between elastography and creatinine

**Fig. 4:**
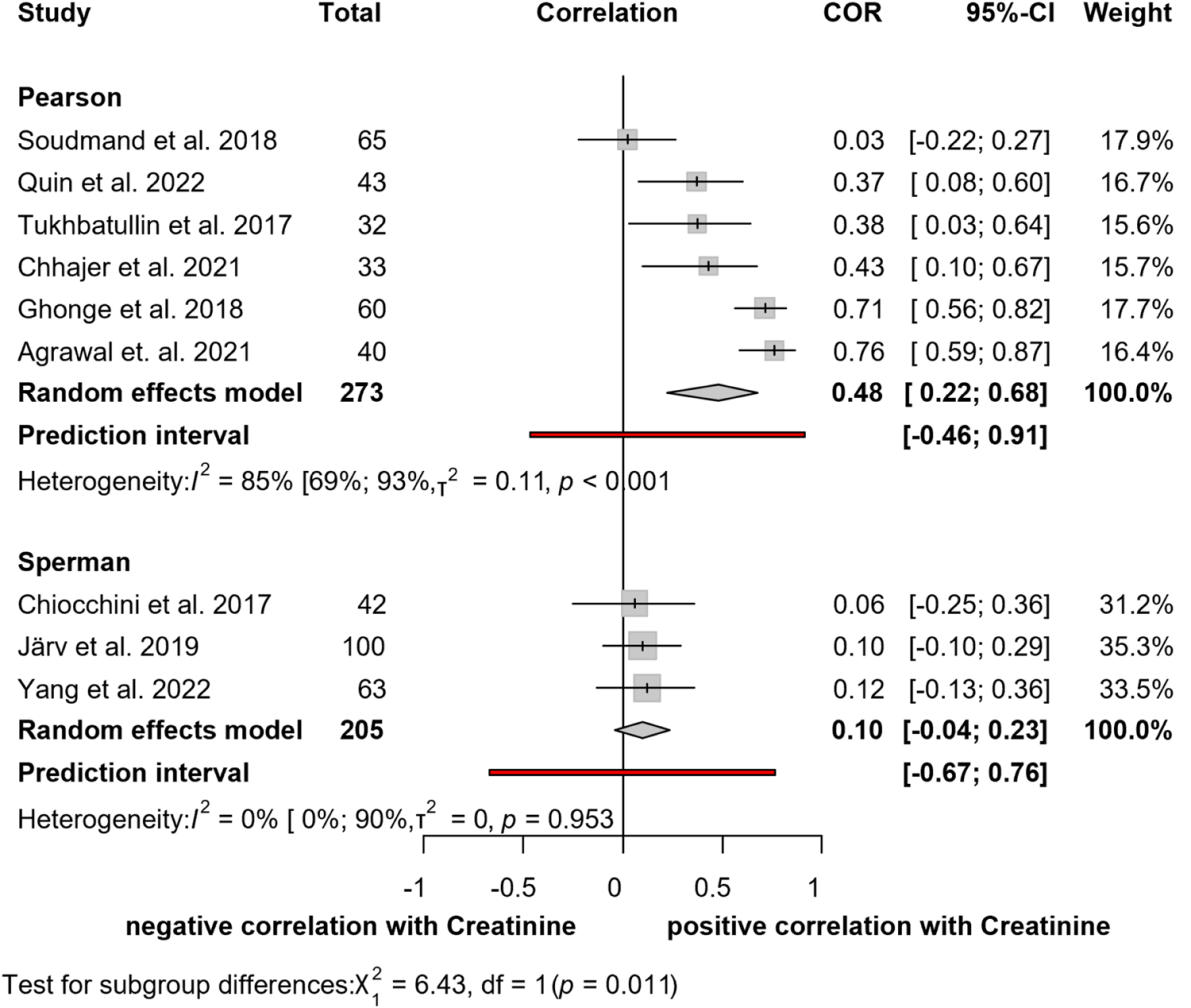
Forest plot of studies representing a moderate positive correlation between creatinine and elastography

The relationship between shear wave elastography and serum creatinine levels was explored in nine studies [10, 15, 25, 26, 29, 32, 34, 37, 38], including 478 patients (Fig. 4). Our results show a moderate positive Pearson's correlation between these two parameters (*r* = 0.48; CI: 0.22, 0.68). Considerable heterogeneity was found between the articles (*I*^*2*^ = 73%; *p* < 0.001). Pooled results for Spearman’s correlation showed negligible correlation (*r* = 0.10; CI:− 0.04, 0.23). No heterogeneity was found between these studies (*I*^2^ = 0%; *p* = 0.953).

#### Correlation between elastography and eGFR

**Fig. 5:**
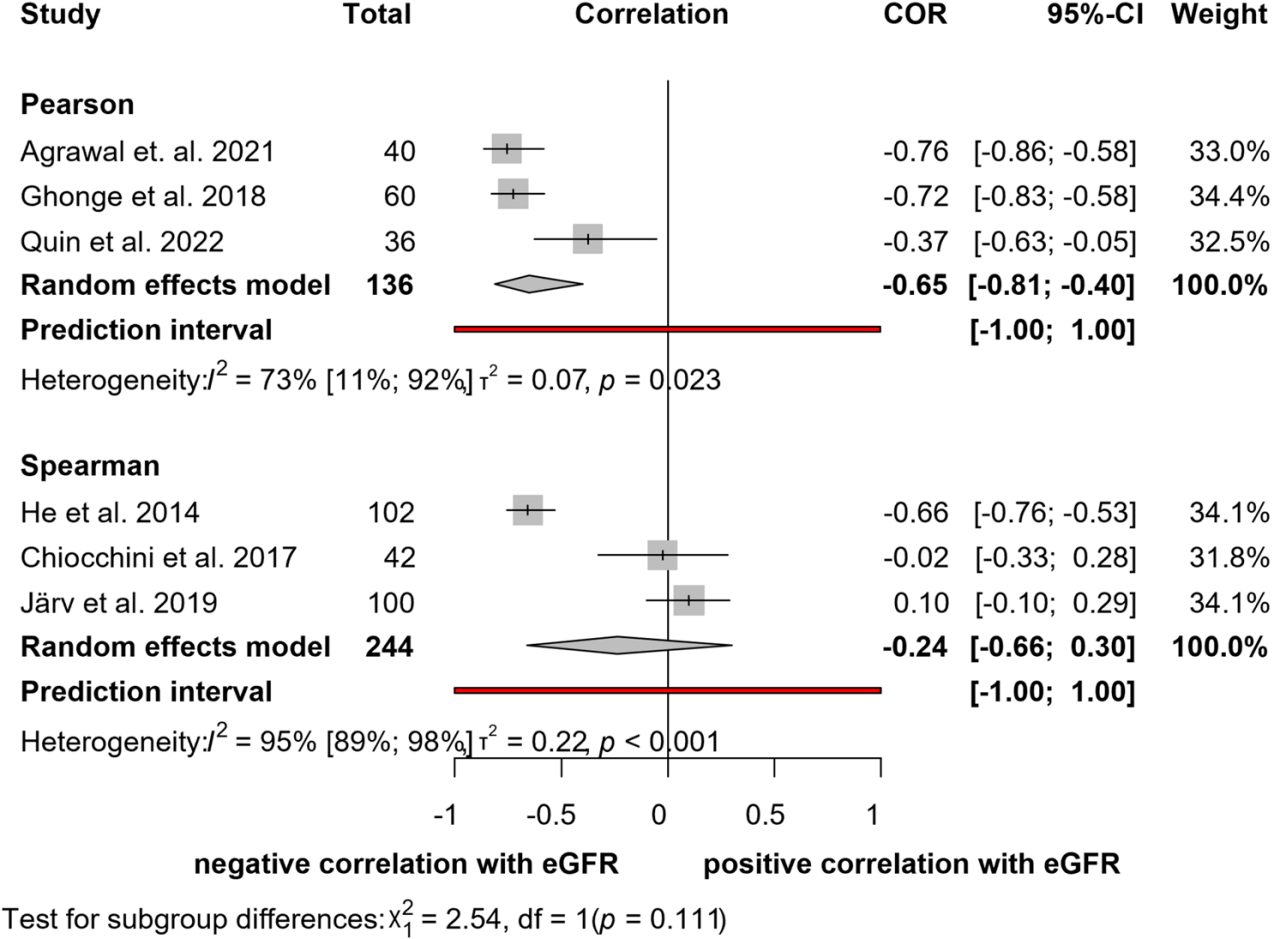
Forest plot of studies representing no correlation between eGFR and elastography

In the case of eGFR, six studies [25, 26, 29, 31, 32, 37] reported calculated correlation coefficients (Fig. 5). The total number of patients was 380. The rate of correlation calculated with pooled Pearson’s correlation coefficient was moderate (*r* = − 0.65; CI: -0.81, 0.40). In this case, heterogeneity was substantial (*I*^2^ = 73%, *p* = 0.023). The results with pooled Spearman's correlation coefficient did not show a statistically significant correlation (*r* = − 0.24; CI: − 0.66, 0.30). The heterogeneity between the studies was significant (*I*^*2*^ = 95%; *p* < 0.001).

The calculation method of eGFR varied throughout the articles. The Modification of Diet in Renal Disease study equation was used in three studies [25, 31, 37], two studies [26, 32] used the Chronic Kidney Disease EPIdemiology formula, and Ghonge et al. [29] applied the Nankivell formula.

## Discussion

In the past decade, the relationship between kidney stiffness measured by elastography and fibrosis has been increasingly investigated. This systematic review and meta-analysis aimed to evaluate the correlation between renal allograft shear wave elastography findings and kidney dysfunction parameters in a kidney transplanted population. Our study showed a positive correlation between kidney allograft elasticity measured by shear wave elastography and kidney biopsy results. Additionally, we found a positive correlation between kidney stiffness and resistive index, a positive correlation between shear wave elastography and creatinine level, and a negative correlation between shear wave elastography and eGFR.

Shear wave elastography has previously been shown to be effective in detecting and measuring the severity of liver fibrosis [39–43]. As transplanted kidneys are more superficially located in the pelvis, shear wave elastography can be used more accurately than in case of native kidneys [44]. He et al. examined 50 patients with stable allograft function and 52 with impaired allograft function and found that the sensitivity and specificity of shear wave elastography to determine allograft dysfunction was 78% and 86.5%, respectively [31]. For the same outcome, Agrawal et al. calculated a sensitivity and specificity of 70.4% and 100%, respectively [25]. Another study by Chhajer et al. examined shear wave elastography to differentiate between low-grade (Banff 0–1) fibrosis and high-grade (Banff 2–3) fibrosis; sensitivity and specificity was 78.9% and 91%, respectively. The ability of shear wave elastography to differentiate grade 2 fibrosis from grade 3 was also tested, with a sensitivity of 83% and specificity of 92% [15].

Ultrasound guidance is an important factor in utilizing shear wave elastography; we focused on this method because of its ease of use and wide availability. However, another possibility for assessing renal fibrosis noninvasively would be magnetic resonance elastography. Magnetic resonance elastography of renal allografts has also been investigated recently [45, 46], but is more expensive and time consuming. On the contrary, ultrasound-guided elastography can be carried out more quickly, without long examination times [47].

Interstitial fibrosis and tubular atrophy are major causes of allograft injury [48]. In the presence of chronic tubulointerstitial damage, the outcome of allograft survival is generally poor [49]. Interstitial fibrosis and tubular atrophy start in the early post-transplant period [2, 50]. In the first year after transplantation, tubulointerstitial damage may develop rapidly and is associated with immunologic factors. This results in irreversible glomerulosclerosis and thus severe impairment of nephrons [2, 50]. Presently, the degree of fibrosis in allografts can only be determined by biopsy, which is an invasive procedure, sampling only < 1% of the kidney [48]. As a semi-quantitative hierarchical classification of chronic lesions, Banff classification often results in interobserver discrepancies [1, 51, 52].

In our study, most of the included articles did not find a strong correlation between fibrosis and kidney stiffness. Based on our results, the lowest correlation rate was found in the study by Desvignes et al.[28] in which only pediatric patients with low fibrosis rates (0–1) were included. A weak correlation was found by Chiocchini et al., who examined patients requiring allograft biopsy for clinical reasons. Additionally, Grenier included patients presenting for protocol biopsies [26, 30]. Furthermore, eight of Stock's patients had histologically-proven rejection the year before the examination [33]. Quin et al. included patients with chronic allograft dysfunction and found one of the highest correlation rates [37]. Data on the number of patients for each Banff grade was available in only two studies. A total of 22.7% of the population in the study by Chhajer et al. had high fibrosis grades (2–3); in Stock et al. this prevalence was 16.7% [15, 33]. Although data are scarce, it is probable that in those studies where the number of patients with fibrosis was higher, the correlation was stronger.

An important tool in the management of allografts is Doppler ultrasound; it determines renal arterial resistive index, which is a semi-quantitative index derived from the evaluation of the renal vasculature [53]. Due to the high vascularization of the kidney, kidney perfusion contributes to mechanical stiffness. The clinical meaning of kidney stiffness measured by shear wave elastography should be interpreted accordingly [54]. There are two theories on how resistive index is significant in determining renal dysfunction. First, the vessels themselves are being injured; alternatively, vessels are influenced by surrounding interstitial fibrosis thus resulting in increased resistive index [55]. Regardless of the cause, ultrasound elastography seems to show renal impairment earlier than Doppler ultrasound [56]. Loock et al. [57] hypothesized that longitudinal resisitive index changes could be more informative than a single measurement of resistive index. Their study showed that in the first year after transplantation, graft loss was significantly more frequent in patients with increasing intrarenal resistive index. Our results showed a moderate positive correlation, but the high heterogeneity in the published data suggests that this association may be incidental. Among our included articles, the population of Ghonge et al. was the most diverse, as they had equal amounts of stable and unstable patients; they also reported the highest rate of correlation [29]. Regarding technical details, the study by Wang et al. was outstanding, as they used a linear transducer and included only transplants within 12 months since transplantation [35]. Although in the study by Soudmand et al. the population consisted of patients with suspected pathology and a high average resistive index, they found no relevant correlation between elastography and Doppler ultrasound [10]. It is also important to point out that resistive index is highly variable as it is influenced by several factors including the patient’s age, hydration status, heart rate, medications, presence and degree of hypertension, hydronephrosis, and other comorbidities [58–60].

Regarding laboratory kidney dysfunction parameters, the highest correlation rate between creatinine and shear wave elastography was found in the two papers by Agrawal et al. and Ghonge et al.; the population in both studies consisted of mainly male patients [25, 29]. Our study found a positive correlation between kidney stiffness and creatinine levels. However, as serum creatinine levels rise in the later phases of allograft failure, they can only be used to predict severe dysfunction [61].

Due to progressive glomerulosclerosis, interstitial fibrosis and tubular atrophy lead to decline of eGFR [61]. This inverse relationship between eGFR and parenchymal stiffness was apparent in our results. A strong correlation rate was found by Ghonge et al., in which allografts with stable and impaired kidney function were studied [29]. One of the inclusion criteria in Chiocchini's study was based on eGFR, but the Authors could not find a significant correlation with parenchymal stiffness [26]. Interestingly, Järv et al. found a significant inverse correlation between shear wave elastography and eGFR. Their population included 100 stable patients, which could explain the discrepancy [32].

To the best of our knowledge, the systematic review and meta-analysis presented herein is the first to assess the correlation between shear wave elastography and kidney dysfunction parameters. With the help of rigorous methodology, we were able to carry out a detailed renal function assessment in a transplanted population.

However, only a few studies could be integrated into our meta-analysis. The populations of the included articles were quite heterogeneous. Exploration of heterogeneity could not be sufficiently carried out because information for subgroup analysis was scarce in the original studies.

Because of their location in the iliac fossa [62], transplanted kidneys lie more superficially than native kidneys, and thus higher-quality images can be acquired by shear wave elastography [31, 44]. Further research is needed to determine the sensitivity and specificity of shear wave elastography to detect fibrosis in the transplanted kidney. The relationship between kidney elasticity and kidney dysfunction parameters such as resistive index, creatinine, and eGFR should be further explored to confirm the reliability of shear wave elastography as an additional tool in renal function assessment. Detailed population data should be reported in future studies.

It is very important for scientific results to be translated into everyday practice [63] therefore, based on our results, we suggest the development of standardized, hardware-specific protocols for the evaluation of allografts with shear wave elastography. Further research is also needed to determine cut-off values for different grades of fibrosis and degrees of allograft dysfunction. Comparative studies comparing shear wave elastography, magnetic resonance elastrography and transplant kidney biopsy could improve our understanding of the association between kidney stiffness and fibrosis.

## Conclusion

In summary, our study found a moderate positive correlation between kidney stiffness measured by shear wave elastography and biopsy results. Noninvasive assessment of kidney fibrosis after transplantation is crucial. However, there is currently insufficient evidence to support elastography over biopsy in the longitudinal management of kidney transplant patients.

## Supplementary Information

Below is the link to the electronic supplementary material.

Supplementary file1 (PDF 579 kb)

## Data Availability

As a systematic review and meta-analysis, data serving as basis for our analysis was extracted from readily available articles in the literature. Type of data extracted is detailed in the methods section of our article. Datasets generated for analysis are available upon request. Original datasets are to be requested from individual studies’ authors.
