# Supplementary material for: Investigating the role of ultrasound-based shear wave elastography in kidney transplanted patients: correlation between non-invasive fibrosis detection, kidney dysfunction and biopsy results—a systematic review and meta-analysis
Source: J Nephrol. 2024 Mar 1;37(6):1509–22. doi: 10.1007/s40620-023-01856-w (PMC11473454; doi:10.1007/s40620-023-01856-w)

Title

Investigating the role of ultrasound-based shear wave elastography in kidney transplanted patients: Correlation of non-invasive fibrosis detection, kidney dysfunction and biopsy results. A systematic review and meta-analysis.

Journal

Journal of Nephrology

Authors

Teodóra Filipov MD[1,2], Brigitta Teutsch MD[2,3,4], Anett Szabó MD[2,5], Attila Forintos[2], Júlia Ács MD[2,5], Alex Váradi MSc[2,6,7], Prof. Péter Hegyi MD, PhD, DSc [2,3,4], Prof. Tibor Szarvas MD, habil., DSc[5,8], Prof. Nándor Ács MD, habil.[9], Prof. Péter Nyirády MD, habil., DSc[5], Pál Ákos Deák MD, PhD[1]

Affiliations:

1. Department of Interventional Radiology, Heart and Vascular Center, Faculty of Medicine, Semmelweis University, Budapest, Hungary
2. Centre for Translational Medicine, Semmelweis University, Budapest, Hungary
3. Institute for Translational Medicine, Medical School University of Pécs, Pécs, Hungary
4. Institute of Pancreatic Diseases, Semmelweis University, Budapest, Hungary
5. Department of Urology, Faculty of Medicine, Semmelweis University, Budapest, Hungary
6. Department of Laboratory Medicine, Medical School, University of Pécs, Pécs, Hungary
7. Department of Metagenomics, University of Debrecen, Debrecen, Hungary
8. Department of Urology, University of Duisburg-Essen and German Cancer Consortium (DKTK)-University Hospital Essen, Essen, Germany
9. Department of Obstetrics and Gynecology, Faculty of Medicine, Semmelweis University, Budapest, Hungary

Corresponding author

Pál Ákos Deák MD, PhD

Postal address: H-1122, Határőr út 18., Budapest, Hungary

Tel.: +36 1 458 6880

E-mail address: deakakos@gmail.com

Table of Contents of the Supplementary Material

Table S1. PRISMA checklist....4

Table S2. Literature search - Search key used in databases....6

Table S3. Assessment of the risk of bias and applicability of included studies for Spearman's correlation between elastography and biopsy results representing a low risk of bias with some uncertainty and no applicability concerns....7

Figure S1. Diagram representing a low risk of bias with some uncertainty and no applicability concerns of included studies for the correlation between elastography and biopsy results assessed by Spearman's correlation....7

Table S4. Assessment of the risk of bias and applicability of included studies for Pearson's correlation between elastography and biopsy results showing low risk....8

Figure S2. Diagram representing a low risk of bias and applicability of included studies for the correlation between elastography and biopsy results assessed by Pearson's correlation....8

Table S5. Assessment of the risk of bias and applicability of included studies for Pearson's correlation between elastography and resistivity index representing a high risk of bias in ''reference standard'' and ''index test'' domains and no applicability concerns....9

Figure S3. Diagram representing a high risk of bias in ''reference standard'' and ''index test'' domains and no applicability concerns of included studies for the correlation between elastography and resistivity index assessed by Pearson's correlation....9

Table S6. Assessment of the risk of bias and applicability of included studies for Spearman's correlation between elastography and resistivity index representing a high risk of bias in ''reference standard'' and ''index test'' domains and no applicability concerns....10

Figure S4. Diagram representing a high risk of bias in ''reference standard'' and ''index test'' domains and no applicability concerns of included studies for the correlation between elastography and resistivity index assessed by Spearman's correlation....10

Table S7. Assessment of the risk of bias and applicability of included studies for Pearson's correlation between elastography and creatinine representing an uncertain risk of bias in ''reference standard'' and ''index test'' domains and no applicability concerns....11

Figure S5. Diagram representing an uncertain risk of bias in ''reference standard'' and ''index test'' domains and no applicability concerns of included studies for the correlation between elastography and creatinine assessed by Pearson's correlation....11

Table S8. Assessment of the risk of bias and applicability of included studies for Spearman's correlation between elastography and creatinine representing an uncertain risk of bias in ''reference standard'' domain and no applicability concerns....12

Figure S6. Diagram representing an uncertain risk of bias in ''reference standard'' and domain and no applicability concerns of included studies for the correlation between elastography and creatinine assessed by Spearman's correlation....12

Table S9. Assessment of the risk of bias and applicability of included studies for Pearson's correlation between elastography and estimated glomerular filtration ratio representing an uncertain risk of bias in ''reference standard'' and “index test” domain and no applicability concerns....13

Figure S7. Diagram representing an uncertain risk of bias in ''reference standard'' and “index test” domain and no applicability concerns of included studies for the correlation between elastography and estimated glomerular filtration ratio assessed by Pearson's correlation 13

Table S10. Assessment of the risk of bias and applicability of included studies for Spearman's correlation between elastography and estimated glomerular filtration ratio representing an uncertain risk of bias in ''reference standard'' domain and no applicability concerns 14

Figure S8. Diagram representing an uncertain risk of bias in ''reference standard'' domain and no applicability concerns of included studies for the correlation between elastography and estimated glomerular filtration ratio assessed by Spearman's correlation 14

Table S1. PRISMA checklist

Section and Topic	Item #	Checklist item	Location where item is reported
**TITLE**			
Title	1	Identify the report as a systematic review.	1
**ABSTRACT**			
Abstract	2	See the PRISMA 2020 for Abstracts checklist.	2
**INTRODUCTION**			
Rationale	3	Describe the rationale for the review in the context of existing knowledge.	3
Objectives	4	Provide an explicit statement of the objective(s) or question(s) the review addresses.	3
**METHODS**			
Eligibility criteria	5	Specify the inclusion and exclusion criteria for the review and how studies were grouped for the syntheses.	5
Information sources	6	Specify all databases, registers, websites, organisations, reference lists and other sources searched or consulted to identify studies. Specify the date when each source was last searched or consulted.	5
Search strategy	7	Present the full search strategies for all databases, registers and websites, including any filters and limits used.	5 & S3
Selection process	8	Specify the methods used to decide whether a study met the inclusion criteria of the review, including how many reviewers screened each record and each report retrieved, whether they worked independently, and if applicable, details of automation tools used in the process.	5
Data collection process	9	Specify the methods used to collect data from reports, including how many reviewers collected data from each report, whether they worked independently, any processes for obtaining or confirming data from study investigators, and if applicable, details of automation tools used in the process.	5
Data items	10a	List and define all outcomes for which data were sought. Specify whether all results that were compatible with each outcome domain in each study were sought (e.g. for all measures, time points, analyses), and if not, the methods used to decide which results to collect.	3-4
	10b	List and define all other variables for which data were sought (e.g. participant and intervention characteristics, funding sources). Describe any assumptions made about any missing or unclear information.	3-4
Study risk of bias assessment	11	Specify the methods used to assess risk of bias in the included studies, including details of the tool(s) used, how many reviewers assessed each study and whether they worked independently, and if applicable, details of automation tools used in the process.	3-4
Effect measures	12	Specify for each outcome the effect measure(s) (e.g. risk ratio, mean difference) used in the synthesis or presentation of results.	3-4
Synthesis methods	13a	Describe the processes used to decide which studies were eligible for each synthesis (e.g. tabulating the study intervention characteristics and comparing against the planned groups for each synthesis (item #5)).	3-4
	13b	Describe any methods required to prepare the data for presentation or synthesis, such as handling of missing summary statistics, or data conversions.	4
	13c	Describe any methods used to tabulate or visually display results of individual studies and syntheses.	5

Section and Topic	Item #	Checklist item	Location where item is reported
	13d	Describe any methods used to synthesize results and provide a rationale for the choice(s). If meta-analysis was performed, describe the model(s), method(s) to identify the presence and extent of statistical heterogeneity, and software package(s) used.	6
	13e	Describe any methods used to explore possible causes of heterogeneity among study results (e.g. subgroup analysis, meta-regression).	6
	13f	Describe any sensitivity analyses conducted to assess robustness of the synthesized results.	6
Reporting bias assessment	14	Describe any methods used to assess risk of bias due to missing results in a synthesis (arising from reporting biases).	5-6
Certainty assessment	15	Describe any methods used to assess certainty (or confidence) in the body of evidence for an outcome.	-
**RESULTS**			
Study selection	16a	Describe the results of the search and selection process, from the number of records identified in the search to the number of studies included in the review, ideally using a flow diagram.	16
	16b	Cite studies that might appear to meet the inclusion criteria, but which were excluded, and explain why they were excluded.	5 & 16
Study characteristics	17	Cite each included study and present its characteristics.	13
Risk of bias in studies	18	Present assessments of risk of bias for each included study.	S7-14
Results of individual studies	19	For all outcomes, present, for each study: (a) summary statistics for each group (where appropriate) and (b) an effect estimate and its precision (e.g. confidence/credible interval), ideally using structured tables or plots.	-
Results of syntheses	20a	For each synthesis, briefly summarise the characteristics and risk of bias among contributing studies.	5 & sup
	20b	Present results of all statistical syntheses conducted. If meta-analysis was done, present for each the summary estimate and its precision (e.g. confidence/credible interval) and measures of statistical heterogeneity. If comparing groups, describe the direction of the effect.	5-6
	20c	Present results of all investigations of possible causes of heterogeneity among study results.	8
	20d	Present results of all sensitivity analyses conducted to assess the robustness of the synthesized results.	-
Reporting biases	21	Present assessments of risk of bias due to missing results (arising from reporting biases) for each synthesis assessed.	sup
Certainty of evidence	22	Present assessments of certainty (or confidence) in the body of evidence for each outcome assessed.	-
**DISCUSSION**			
Discussion	23a	Provide a general interpretation of the results in the context of other evidence.	6-8
	23b	Discuss any limitations of the evidence included in the review.	8
	23c	Discuss any limitations of the review processes used.	8

Section and Topic	Item #	Checklist item	Location where item is reported
	23d	Discuss implications of the results for practice, policy, and future research.	8
**OTHER INFORMATION**			
Registration and protocol	24a	Provide registration information for the review, including register name and registration number, or state that the review was not registered.	3
	24b	Indicate where the review protocol can be accessed, or state that a protocol was not prepared.	3
	24c	Describe and explain any amendments to information provided at registration or in the protocol.	-
Support	25	Describe sources of financial or non-financial support for the review, and the role of the funders or sponsors in the review.	2
Competing interests	26	Declare any competing interests of review authors.	-
Availability of data, code and other materials	27	Report which of the following are publicly available and where they can be found: template data collection forms; data extracted from included studies; data used for all analyses; analytic code; any other materials used in the review.	-

Table S2. Literature search - Search key used in databases

Pubmed	(kidney OR renal) AND (transplant OR graft OR recipients OR allograft) AND (elasticity OR elastograph* OR "shear-wave" OR "shear wave" OR SWE OR ultrasonograph* OR ultrasound OR acoustic OR ARFI) AND (dysfunction OR stiffness OR fibrosis OR structure OR parenchyma)
Central	(kidney OR renal) AND (transplant OR graft OR recipients OR allograft) AND (elasticity OR elastograph* OR "shear-wave" OR "shear wave" OR SWE OR ultrasonograph* OR ultrasound OR acoustic OR ARFI) AND (dysfunction OR stiffness OR fibrosis OR structure OR parenchyma)
Embase	(kidney OR renal) AND (transplant OR graft OR recipients OR allograft) AND (elasticity OR elastograph* OR 'shear-wave' OR 'shear wave' OR SWE OR ultrasonograph* OR ultrasound OR acoustic OR ARFI) AND (dysfunction OR stiffness OR fibrosis OR structure OR parenchyma)

Table S3. Assessment of the risk of bias and applicability of included studies for Spearman's correlation between elastography and biopsy results representing a low risk of bias with some uncertainty and no applicability concerns

STUDY	RISK OF BIAS				APPLICABILITY CONCERNS		
	**Patient selection**	**Index test**	**Reference standard**	**Flow and timing**	**Patient selection**	**Index test**	**Reference standard**
Desvignes et al. 2021	☺	☺	☺	☺	☺	☺	☺
Chiocchini et al. 2017	☺	☺	☺	☺	☺	☺	☺
Stock et al. 2010	☺	☺	?	☺	☺	☺	☺
Barsoum et al. 2022	☺	?	?	☺	☺	☺	☺
Quin et al. 2022	☺	☺	☺	☺	☺	☺	☺
Dai et al. 2014	☺	?	?	?	☺	☺	☺

☺ Low Risk ☹ High Risk ? Unclear Risk

Figure S1. Diagram representing a low risk of bias with some uncertainty and no applicability concerns of included studies for the correlation between elastography and biopsy results assessed by Spearman's correlation

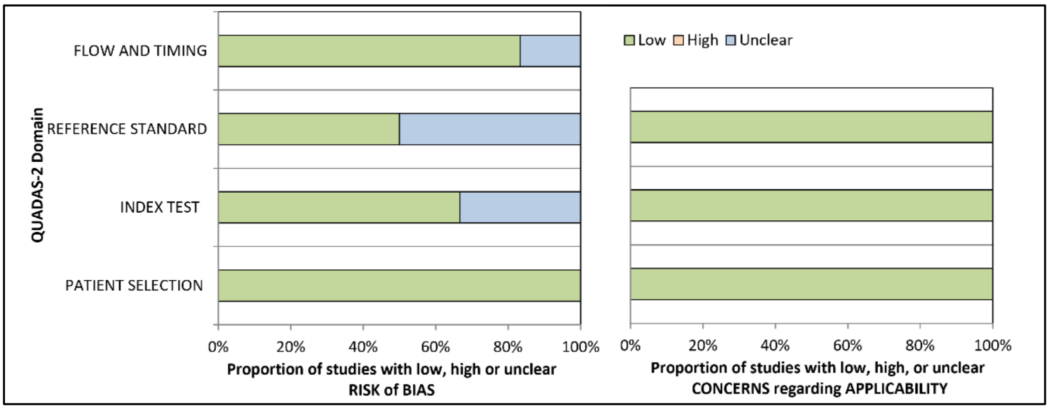

Table S4. Assessment of the risk of bias and applicability of included studies for Pearson's correlation between elastography and biopsy results showing low risk

STUDY	RISK OF BIAS				APPLICABILITY CONCERNS		
	Patient selection	Index test	Reference standard	Flow and timing	Patient selection	Index test	Reference standard
Gernier et.al. 2012	☺	☺	☺	☺	☺	☺	☺
Soudmand et al. 2018	☺	☺	☺	☺	☺	☺	☺
Chhajer et al. 2021	☺	☺	☺	☺	☺	☺	☺

☺Low Risk ☹High Risk ? Unclear Risk

Figure S2. Diagram representing a low risk of bias and applicability of included studies for the correlation between elastography and biopsy results assessed by Pearson's correlation

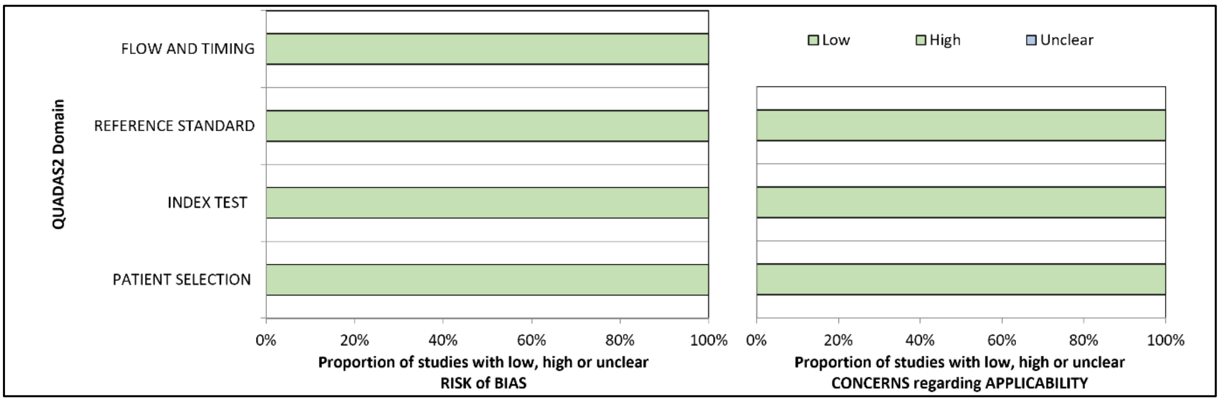

Table S5. Assessment of the risk of bias and applicability of included studies for Pearson's correlation between elastography and resistivity index representing a high risk of bias in "reference standard" and "index test" domains and no applicability concerns

STUDY	RISK OF BIAS				APPLICABILITY CONCERNS		
	Patient selection	Index test	Reference standard	Flow and timing	Patient selection	Index test	Reference standard
Soudmand et al. 2018	☺	☹	☹	☺	☺	☺	☺
Quin et al. 2022	☺	☹	☹	☺	☺	☺	☺
Wang et al. 2017	☺	☹	☹	☺	☺	☺	☺
Agrawal et.al. 2021	☺	☹	☹	☺	☺	☺	☺
Ghonge et al. 2018	☺	☹	☹	☺	☺	☺	☺

☺Low Risk ☹High Risk ? Unclear Risk

Figure S3. Diagram representing a high risk of bias in "reference standard" and "index test" domains and no applicability concerns of included studies for the correlation between elastography and resistivity index assessed by Pearson's correlation

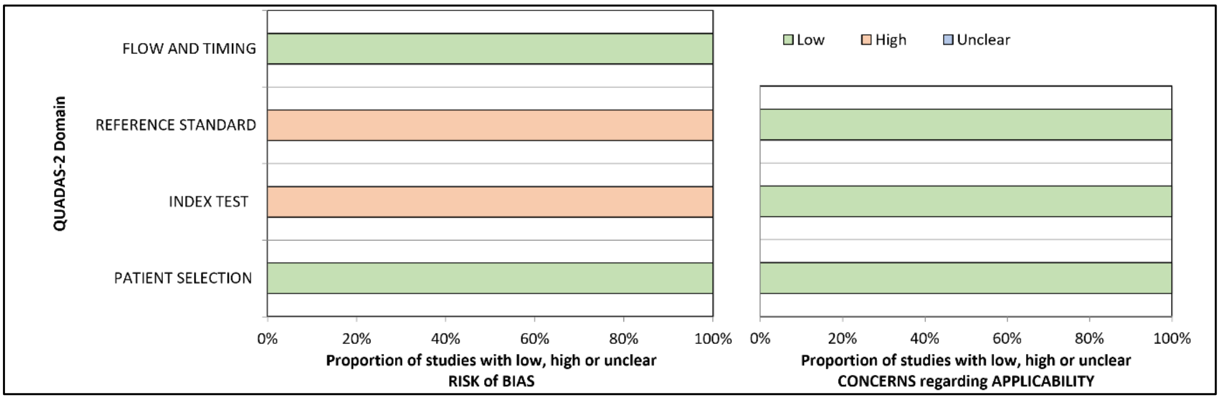

Table S6. Assessment of the risk of bias and applicability of included studies for Spearman's correlation between elastography and resistivity index representing a high risk of bias in "reference standard" and "index test" domains and no applicability concerns

STUDY	RISK OF BIAS				APPLICABILITY CONCERNS		
	Patient selection	Index test	Reference standard	Flow and timing	Patient selection	Index test	Reference standard
Chiocchini et al. 2017	☺	☹	☹	☺	☺	☺	☺
Stock et al. 2010	☺	☹	☹	☺	☺	☺	☺
Yang et al. 2022	☺	☹	☹	☺	☺	☺	☺

☺ Low Risk ☹ High Risk ? Unclear Risk

Figure S4. Diagram representing a high risk of bias in "reference standard" and "index test" domains and no applicability concerns of included studies for the correlation between elastography and resistivity index assessed by Spearman's correlation

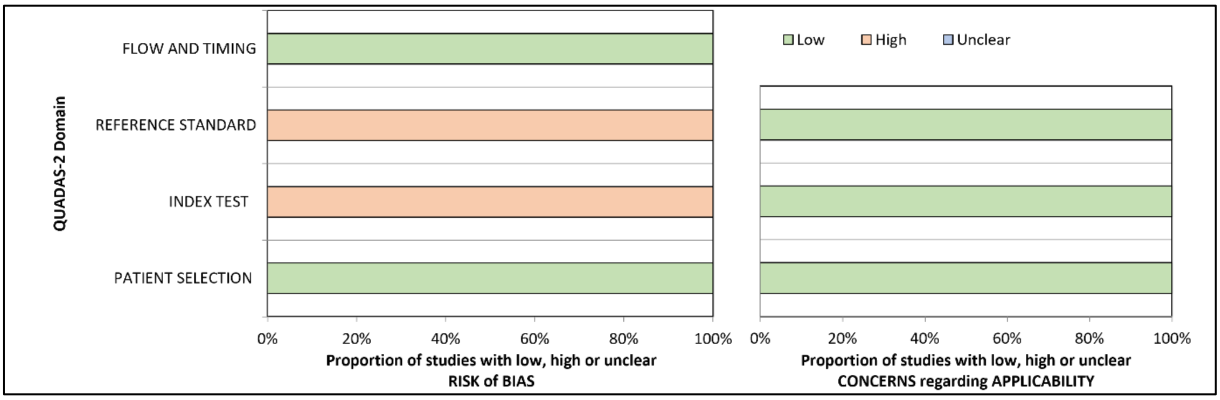

Table S7. Assessment of the risk of bias and applicability of included studies for Pearson's correlation between elastography and creatinine representing an uncertain risk of bias in "reference standard" and "index test" domains and no applicability concerns

STUDY	RISK OF BIAS				APPLICABILITY CONCERNS		
	Patient selection	Index test	Reference standard	Flow and timing	Patient selection	Index test	Reference standard
Soudmand et al. 2018	☺	?	?	☺	☺	☺	☺
Quin et al. 2022	☺	?	?	☺	☺	☺	☺
Tukhbatullin et al. 2017	☺	?	?	☺	☺	☺	☺
Chhajer et al. 2021	☺	☺	?	☺	☺	☺	☺
Ghonge et al. 2018	☺	☺	?	☺	☺	☺	☺
Agrawal et.al. 2021	☺	?	?	☺	☺	☺	☺

☺Low Risk ☹High Risk ? Unclear Risk

Figure S5. Diagram representing an uncertain risk of bias in "reference standard" and "index test" domains and no applicability concerns of included studies for the correlation between elastography and creatinine assessed by Pearson's correlation

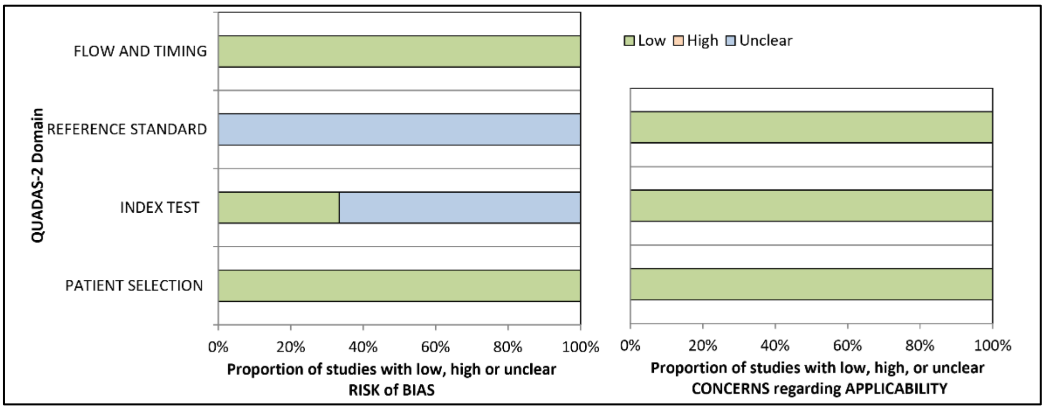

Table S8. Assessment of the risk of bias and applicability of included studies for Spearman's correlation between elastography and creatinine representing an uncertain risk of bias in "reference standard" domain and no applicability concerns

STUDY	RISK OF BIAS				APPLICABILITY CONCERNS		
	Patient selection	Index test	Reference standard	Flow and timing	Patient selection	Index test	Reference standard
Chiocchini et al. 2017	☺	☺	?	☺	☺	☺	☺
Järv et al. 2019	☺	☺	?	☺	☺	☺	☺
Yang et al. 2022	☺	?	?	☺	☺	☺	☺

☺Low Risk ☹High Risk ? Unclear Risk

Figure S6. Diagram representing an uncertain risk of bias in "reference standard" and domain and no applicability concerns of included studies for the correlation between elastography and creatinine assessed by Spearman's correlation

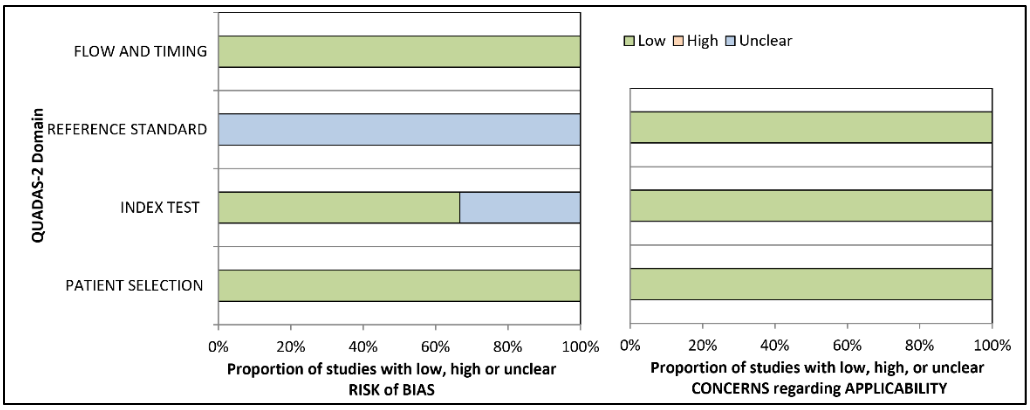

Table S9. Assessment of the risk of bias and applicability of included studies for Pearson's correlation between elastography and estimated glomerular filtration ratio representing an uncertain risk of bias in "reference standard" and “index test” domain and no applicability concerns

STUDY	RISK OF BIAS				APPLICABILITY CONCERNS		
	Patient selection	Index test	Reference standard	Flow and timing	Patient selection	Index test	Reference standard
Agrawal et al. 2021	☺	?	?	☺	☺	☺	☺
Chiocchini et al. 2017	☺	☺	?	☺	☺	☺	☺
Quin et al. 2022	☺	?	?	☺	☺	☺	☺

☺Low Risk ☹High Risk ? Unclear Risk

Figure S7. Diagram representing an uncertain risk of bias in "reference standard" and “index test” domain and no applicability concerns of included studies for the correlation between elastography and estimated glomerular filtration ratio assessed by Pearson's correlation

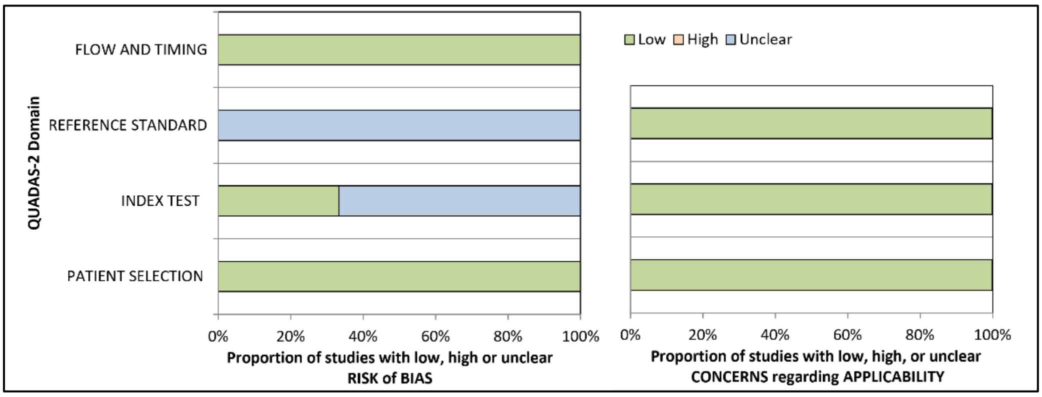

Table S10. Assessment of the risk of bias and applicability of included studies for Spearman's correlation between elastography and estimated glomerular filtration ratio representing an uncertain risk of bias in "reference standard" domain and no applicability concerns

STUDY	RISK OF BIAS				APPLICABILITY CONCERNS		
	Patient selection	Index test	Reference standard	Flow and timing	Patient selection	Index test	Reference standard
He et al. 2014	☺	☺	?	☺	☺	☺	☺
Chiocchini et al. 2017	☺	☺	?	☺	☺	☺	☺
Järv et al. 2019	☺	☺	?	☺	☺	☺	☺

☺Low Risk ☹High Risk ? Unclear Risk

Figure S8. Diagram representing an uncertain risk of bias in "reference standard" domain and no applicability concerns of included studies for the correlation between elastography and estimated glomerular filtration ratio assessed by Spearman's correlation

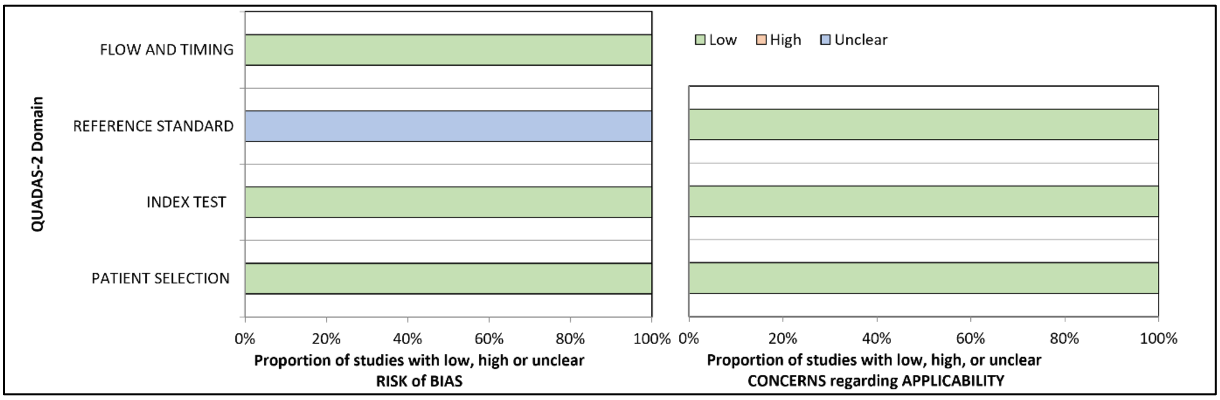

Supplement: Supplementary file 1 — Supplementary file1 (PDF 579 kb) [file 40620_2023_1856_MOESM1_ESM.pdf]
